# Vagitus Uterinus

**Published:** 1913-01

**Authors:** 


### EDITORIAL DEPARTMENT.

DR. F. E. DANIEL, Editor DR. R. H. L. BIBB, Associate Editor

DEPARTMENTS { EUGENICS: Drs. M. Duggan and T. Y. Hull.

WOMAN'S DEPARTMENT: Mrs. F. E. Daniel.

### Vagitus Uterinus.

Of recent this subject has been somewhat extensively discussed in French and Belgian medical journals, and as the intrauterine noises which so much resemble the muffled cries of new-born babes, and which seem to have been met with mostly in cases requiring obstetrical operations or manipulations, have been attributed rather to the inrush or outrush of air incident to manipulative intervention on the part of the *"partero,"* or to fibrillar contractions of a flabby uterus, than to baby-cries, the subject is not altogether void of interest, especially since some competent observers report cases which seem to establish the fact of a true *"vagitus uterinus."*

Mention is made by Tarnier and Chantreuil, in "Modifications of the Fetal Functions Produced by Labor," published in 1888, of intrauterine cries, but Velpeau is reported to have said: "If I had heard them myself, I should not have believed in them." And so the matter rested.

Several years ago Sippel (*British Medical Journal,* September 14, 1912) reported a case in which version was done. A severe contraction, which did not expel his hand, obliged him to stop. Immediately a high-pitched sound was heard within the patient's abdomen resembling an infant's cry; but he noted that, with each successive cry, a current of air ran along his arm, producing a series of vibrations in a fold of mucous membrane which surrounded his forearm, to which occurrence he attributed the *"vagitus,"* instead of to the baby.

Allard reports (*Normandie Medicale,* epitomized in the *British Medical Journal,* July 20, 1912): After an unsuccessful application of the forceps whilst allowing his patient to rest, he heard cries, which were very distinct, though muffled, like the crying of a newly born babe under bedclothes. A nurse who was holding the patient's right remarked that the baby was evidently growing weary and impatient, as he was crying, and almost at the same moment the mother inquired what the noise was. The husband, also a physician, who was behind the nurse, also heard the sounds, which were repeated four or five times successively, and were followed by a set of cries which could be heard all over the room. The child was a vigorous one, and violent movements could be felt over the abdomen; during the last three months of pregnancy the mother suffered much from the fetal movements. The child was delivered dead, either from inspiration of the liquor amnii or from compression during extraction, and could not be resuscitated. No autopsy was made, and thus a valuable opportunity for anatomical demonstration of the child's lungs having functionated and thus prove the fact of *"vagitus uterinus"* was lost.

Basing his opinion on the facts observed by himself in this case, Allard declares that had Velpeau been present he would have been convinced, and that intrauterine crying, however rare, may happen, as he can prove by the four disinterested persons present at this confinement.

Dr. J. Reidy (*British Medical Journal,* October 26, 1912) reports Mrs. C., secundipara, was found with the os fully dilated, membranes protruding, head not engaged; the membranes were ruptured, a leg seized and internal version performed. During this manipulation, the child cried *"in utero,"* much to the alarm of the mother and of the nurse in attendance. He also reports a case of *vagitus vaginitus* in the same article, the two cases comprising his experience in 3000 midwifery cases.

In the same publication, Dr. Oliver Smithson reports that in an occipito-posterior forceps case, immediately upon application of the instruments, and subsequently, the child's cry was distinctly heard by himself, the mother and nurse. He also writes that a few days before this experience, his partner, Dr. Robert D. Bell, told him that a few nights before he had heard intrauterine crying in the woman he assisted the night before, much to his own and to the attending nurse's surprise, as well as to the surprise of several women in the room at the time. Dr. Smithson, with Dr. Allard, thinks that, had Velpeau been present, he would have changed his mind and would have become a firm believer in intrauterine crying.

Mr. A. Teevan (same journal) reports that in an induced labor, at the eighth month, wherein he had passed a female catheter well up between the membranes and the uterine wall, the former were pierced, permitting escape of the amniotic fluid, in which the babe, soon after, cried several times, and as loud as if it had been born, much to the consternation of himself, the mother and the nurse. The labor terminated happily for both the mother and the child.

In 1898 (*British Medical Journal,* May 16) Dr. Kevin reported the case of a child, in his practice, which had cried and sobbed for two hours before birth. The membranes had ruptured and Dr. Kevin could distinctly feel the apex of the child's left ear by sweeping the finger around the os. It was after one of these manipulations that the crying and sobbing began.

A few years ago, in applying the forceps in a second labor, I distinctly heard the baby cry, as did a half dozen of the patient's family who were in the room. The membranes had ruptured, the waters had escaped and the head was engaging. In passing the male blade of the forceps, the child's cry was so distinct that its grandmother declared I was murdering it and ordered me to desist. I did neither, but delivered speedily and without accident.

An analysis of the cases herein mentioned discloses the fact that in all of them the manipulations to which they were subjected were of such a nature as to insure the entrance of air into the uterus and in contact with the baby, without which crying would be impossible, and it is just as rational to attribute the cries to the babe *in utero* as it is to the in and outrush of air, or to the vibrations of a fold of mucous membrane encircling the physician's forearm. Take the case reported by Dr. Reidy, with an experience of 3000 confinements, from which he certainly had learned enough to recognize the cry of a baby whenever and wherever he might hear it, not to write of Dr. Kevin's case, wherein the baby "had cried and sobbed for two hours before birth," nor to mention my own, based upon some 600 cases of midwifery, in which the *vagitus* was as unmistakable as any I ever heard.

Whilst not of much, if of any, practical importance, the subject is interesting, and I will be grateful to any one of like experience who will kindly furnish me with the facts therein.

R. H. L. BIBB.

---

I TAKE OFF MY HAT to this police chief. He here echoes what the RED BACK has been preaching twenty-five years! A drunken man is a sick man—he is poisoned—and the hospital, and not the jail, is the place for him. How much longer will an enlightened (?) people license men to intoxicate the laboring men, and then punish them for being intoxicated? Remove the cause, and the effect will cease.—Ed.

Houston, Texas, December 24.—Chief of Police E. Cullen Noble announced a few days ago that he wanted to give 100 poor families a sober husband and father for a Christmas present. He established a "municipal jag school," put Dr. J. J. McKenna of

Oklahoma City at the head of it and invited all wives, sons and daughters of inebriate husbands and fathers to take them to the police station for treatment.

"When I administer charity, it must be substantial charity," said Chief Noble. "Instead of sending turkey and cranberries to homes that have been reduced to poverty and had the husband or father stolen away by drink, I want to install in these homes a sober and reliable breadwinner. I don't want to tease the appetites of the poor unfortunates with delicious edibles just because it is Christmas, and let them go without breakfast the following morning.

"More sober and reliable husbands is the crying need of wrecked families throughout the world."

The "municipal jag school" met with such success that Chief Noble announced four days later it would become a permanent branch of the police department, and no more "drunks" would be fined.

Now, Police Judge John A. Kirlicks neither fines nor sentences inebriates to jail. Instead he says: "I find the defendant guilty and sentence him (or her) to one course of treatment in the 'municipal jag school.' "

Since Chief Noble announced that he would give away "sober husbands," broken-hearted wives, sons, daughters, mothers and sweethearts have taken their inebriate loved ones to the station and enrolled them in "jag classes."

Within three days after the invitation was issued, twenty-five heavy-eyed and remorseful down-and-outs were taking the treatment. They stay at the station three days, and on leaving are given enough medicine to last thirty days.

The first twenty-five men have been pronounced cured and graduated into the Houston municipal employment bureau, and from there sent out on jobs. Chief Noble personally conducts the employment bureau.

None of the twenty-five men had jobs when they entered the classes. They are required to report to Chief Noble, and so far none have fallen from the wagon.

"Habitual drinking is not a vice—it is a disease," says Chief Noble. "To fine a man for his affliction is a crime. They should never be jailed, except to subdue them when they are violent. Every city should have an inebriate hospital.

"Every time one of these afflicted men is locked up or fined, he is struck a discouraging blow, and some wife or child at home suffers a little more.

"Society has too long and foolishly tried to wipe out this dread evil with jails and lockups. As long as I am chief of police, Houston will reclaim her poor alcohol victims."

Dr. McKenna says he can convert 100 Houston drinkers into sober breadwinners. By that time, he thinks, the work of the school will become lighter, but he says it should never be abolished.

